# Protocol summary and statistical analysis plan for the bone loss prevention with zoledronic acid or denosumab in critically ill adults (BONE ZONE) trial^☆^

**DOI:** 10.1016/j.ccrj.2026.100173

**Published:** 2026-03-24

**Authors:** Neil Orford, Ary Serpa Neto, Priya Nair, Allison Bone, Jacqueline R. Center, Carol Hodgson, Mark A. Kotowicz, Edward Litton, Claire Reynolds, Tony Trapani, Paul Young, Balasubramanian Venkatesh, the Bone Zone Investigators, the ANZICS Clinical Trials Group

**Affiliations:** a University Hospital Geelong, Barwon Health, Geelong, Victoria, Australia; b School of Medicine, Deakin University, Geelong, Victoria, Australia; c Australian and New Zealand Intensive Care Research Centre (ANZIC-RC), School of Public Health and Preventative Medicine (SPHPM), Monash University, Melbourne, Victoria, Australia; d Department of Critical Care, University of Melbourne, Melbourne, Victoria, Australia; e Department of Critical Care Medicine, Hospital Israelita Albert Einstein, São Paulo, Brazil; f Critical Care Division, The George Institute for Global Health, University of New South Wales, Sydney, Australia; g St Vincent's Hospital Sydney, Darlinghurst, New South Wales, Australia; h Gold Coast University Hospital, Southport, Queensland, Australia; i Department of Intensive Care, Austin Hospital, Melbourne Victoria, Australia; j Faculty of Medicine, University of New South Wales, Sydney, New South Wales, Australia; k Department of Intensive Care, The Alfred Hospital, Melbourne, Victoria, Australia; l Bone Biology Division, Garvan Institute of Medical Research, Sydney, New South Wales, Australia; m Intensive Care Unit, Fiona Stanley Hospital, Robin Warren Drive, Murdoch, Western Australia, Australia; n School of Medicine, University of Western Australia, Crawley, Western Australia, Australia; o Department of Intensive Care, Wellington Regional Hospital, Wellington, New Zealand; p Medical Research Institute of New Zealand, Wellington, New Zealand; q Deakin University, IMPACT (Institute for Mental and Physical Health and Clinical Translation), Geelong, Australia; r Department of Medicine-Western Health, The University of Melbourne, St Albans, Australia; s NHMRC Clinical Trials Centre, University of Sydney, St Vincent's Hospital, Sydney, Australia

**Keywords:** Protocol, Statistical analysis plan, Bayesian, Intensive care, Osteoporosis, Clinical trial, Bone antiresorptive therapy

## Abstract

**Background:**

Older critically ill women and men experience accelerated bone loss, an increased risk of fragility fracture, and the associated burden of impaired quality of life and increased risk of death. Antiresorptive therapies are commonly used, safe, and effective in preventing bone loss in ambulatory patients. There is insufficient evidence for their routine use during recovery from critical illness.

**Objective:**

The objective of this study was to describe the trial protocol and statistical analysis plan for Bone Loss Prevention with Zoledronic Acid or Denosumab in Critically Ill Adults (BONE ZONE) trial.

**Design, setting, participants, and intervention:**

A phase IIb, multicentre, prospective, investigator-initiated, double-blind, placebo-controlled, randomised trial, with a target sample size of 330 participants. Women aged 50 years or older and men aged 70 years or older recovering from critical illness are randomly assigned to receive zoledronic acid, denosumab, or placebo in a 1:1:1 ratio.

**Main outcome measures:**

The primary outcome is annualised change in femoral neck bone mineral density in the year after intensive care unit discharge**.** Secondary outcomes include change in lumbar spine bone mineral density, fracture incidence, falls, hospital readmissions, mortality, safety outcomes, and quality of life. All analyses will be conducted on an intention to treat basis.

**Conclusion:**

The BONE ZONE trial will evaluate the efficacy and safety of antiresorptive medication administered during recovery from critical illness to patients at higher risk of bone loss and provide robust evidence to inform the need and design for a phase III trial.

## 1 Introduction

Patients admitted to an intensive care unit (ICU) face health issues that extend beyond their critical illness. Accelerated bone loss is a specific risk following critical illness, beginning in the first days of critical illness and being greatest in women aged 50 years or older and men aged 70 years or older.[1], [2], [3] One year after critical illness, 80% of women aged 50 years or older are classified as osteoporotic or osteopaenic, and both women and men have a significantly greater decrease in bone mineral density (BMD) than age-adjusted population controls.^1^ In subsequent years, older women who survive critical illness have a higher fragility fracture rate compared with community age-matched controls.^2^

Bone antiresorptive therapies are effective in reducing bone loss and decreasing fracture risk. Zoledronic acid and denosumab are bone antiresorptive therapies with established efficacy in non-critically ill populations and can be administered during recovery from critical illness.[4], [5], [6], [7] In addition to their skeletal effects, these agents are associated with reduced mortality.^5^^,^[8], [9], [10]

There is insufficient high-quality evidence to support the routine use of antiresorptive medications during recovery from critical illness.^6^^,^^7^^,^[10], [11], [12], [13] The Bone Loss Prevention with Zoledronic Acid or Denosumab in Critically Ill Adults (BONE ZONE) trial will evaluate the efficacy and safety of zoledronic acid and denosumab for prevention of BMD loss in the year after critical illness in women aged 50 years or older and men aged 70 years or older requiring ICU admission for more than two calendar days.

## 2 Methods

### 2.1 Trial design

**Fig. 1:**
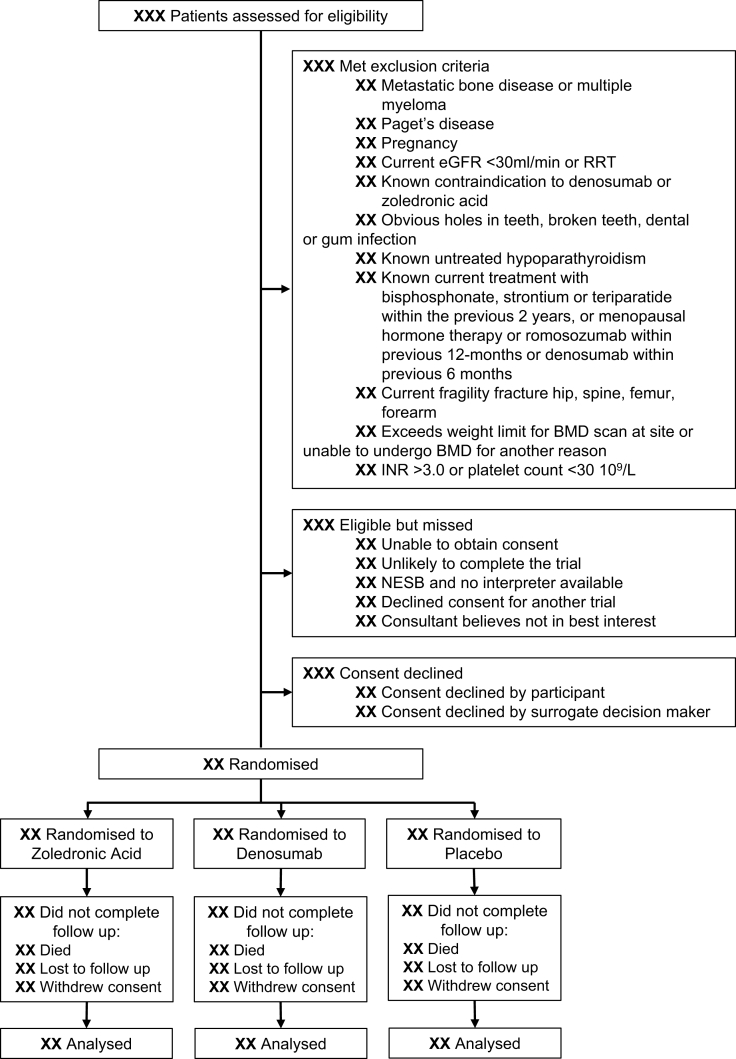
Proposed reporting of the flow of participants through the trial. Abbreviations: BMD = bone mineral density; eGFR = estimated glomerular filtration rate; INR = international normalised ratio; NESB = non-English-speaking background; RRT = renal replacement therapy.

BONE ZONE is a phase IIb, investigator-initiated, multicentre, prospective, double-blind, placebo-controlled, randomised trial designed to test the hypothesis that administration of zoledronic acid or denosumab during recovery from critical illness will effectively and safely attenuate critical illness–associated loss of BMD in the year after critical illness (Fig. 1). The trial is designed with reference to the Standard Protocol Items: Recommendations for Interventional Trials checklist.^14^ The trial design is informed by the findings of a pilot trial reporting feasibility with respect to patient selection, protocol compliance, safety, and recruitment.^7^ The protocol is approved primarily by the Austin Health Human Research Ethics Committee (HREC/69234Austin 2020, version 7, 22 July 2025). Consumers are involved in all aspects of trial design and conduct and are represented on the trial management committee. The trial is prospectively registered with the Australian New Zealand Clinical Trials Registry (ACTRN12621000085875) and Clinicialtrials.gov (NCT04608630).

### 2.2 Setting and population

The trial is being conducted at 24 ICUs in Australia and New Zealand. Recruitment commenced in July 2021. Women aged 50 years or older and men aged 70 years or older who have been in ICU for two or more calendar days and are receiving ICU-level support and not meeting exclusion criteria will be eligible for inclusion (Table 1). A hierarchical approach to consent is used, consistent with the National Health and Medical Research Council *National Statement on Ethical Conduct in Human Research*.^15^

**Table 1:** Eligibility criteria.

Inclusion criteria	1. Women aged ≥50 years or men aged ≥70 years 2. In ICU for 2 or more calendar days and not expected to be discharged on the second day 3. Required ICU-level support (i.e. intravenous vasoactive drugs, or invasive mechanical ventilation, or non-invasive ventilation, or high-flow nasal oxygen at FiO_2_≥0.4 and/or gas flows ≥40L/min) for a minimum cumulative duration of 6 h 4. Expected to survive the current hospital admission
Exclusion criteria	1. Cancer-related metastatic bone disease or multiple myeloma 2. Paget's disease 3. Pregnancy 4. Current eGFR <30 LmL/min or receiving renal replacement therapy 5. Known contraindication to denosumab or zoledronic acid 6. Obvious holes in teeth or broken teeth or dental/gum infection 7. Known untreated hypoparathyroidism 8. Known current treatment with bisphosphonate, strontium or teriparatide within previous 2 years, or menopausal hormone therapy or romosozumab within the previous 12 months or denosumab within previous 6 months 9. Current fragility fracture of the hip, spine, femur, or forearm 10. Exceeds weight limit for BMD scan at site or unable to undergo BMD for another reason 11. INR>3.0 or Platelet count <30 × 10^9^/L

Abbreviations: BMD = bone mineral density; eGFR = estimated glomerular filtration rate; FiO_2_ = fraction of inspired oxygen; ICU = intensive care unit; INR = international normalised ratio; RRT = renal replacement therapy.

### 2.3 Randomisation, blinding, and allocation concealment

Participants are randomised on a 1:1:1 basis to zoledronic acid, or denosumab, or placebo. A permuted-block randomisation method with variable block sizes, stratified by site and sex at birth, is used to allocate eligible participants to each of the three trial treatments, using independent statistician–formulated, computer-driven, randomly allocated sequence generation.

Following randomisation, unblinded staff members prepare trial medication according to the allocated treatment. The allocation sequence is unknown to the unblinded staff preparing trial medication, clinicians, investigators, and statistician conducting the analyses. All personnel, apart from unblinded staff, are blinded to treatment allocation. The unblinded staff members are not involved in trial participant care, entry of outcome data, or statistical analysis.

### 2.4 Trial treatments

Participants receive zoledronic acid (a single dose of 5 mg intravenously) or denosumab (60 mg subcutaneously every 6 months) or placebo (0.9% sodium chloride or 5% dextrose) according to treatment allocation. Trial medication is prepared as blinded zoledronic acid/placebo trial formulation (100 mL bag 0.9% sodium chloride or 5% dextrose or zoledronic acid), blinded denosumab/placebo trial formulation in a 3-mL syringe (placebo 0.9% sodium chloride or denosumab). Zoledronic acid/placebo trial formulation is administered as an intravenous infusion via existing vascular access over at least 15 min. Denosumab/placebo trial formulation is administered as a room-temperature subcutaneous injection.

Trial medication is administered when the participant is assessed as having no new or untreated sepsis, afebrile for the previous 24 h (temperature <38 °C), and receiving no or minimal vasopressor support (noradrenaline <10 mcg/min). On day 0, participants receive both zoledronic acid/placebo trial formulation and denosumab/placebo trial formulation. On day 180, participants receive denosumab/placebo trial formulation only.

**Fig. 2:**
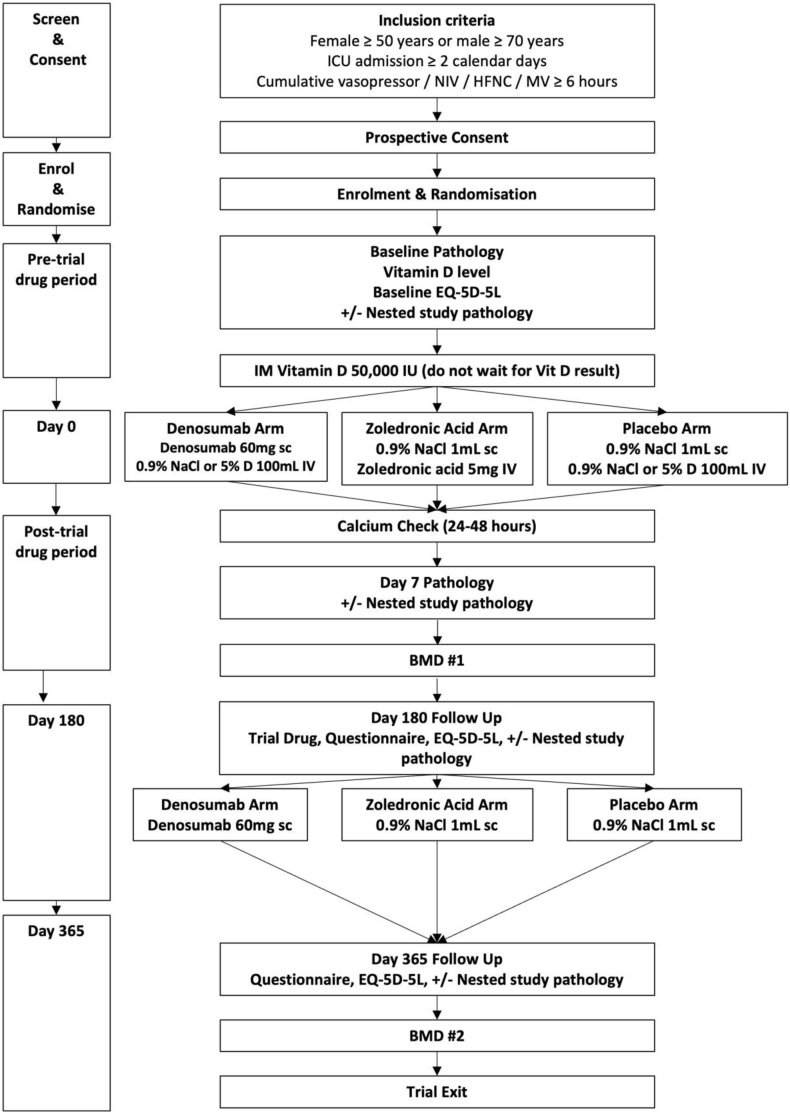
Study schedule for patients included in BONE ZONE. Abbreviations: BMD = bone mineral density; D = dextrose; EQ-5D-5L = EuroQol 5 Dimensions 5 Levels; HFNC = high-flow nasal cannula; ICU = intensive care unit; IU = international units; IV = intravenous; MV = mechanical ventilation; NaCl = sodium chloride; NIV = non-invasive ventilation; sc = subcutaneous.

Baseline serum vitamin D (25-hydroxy-D) is collected and cholecalciferol 50 000 IU is administered as an intramuscular injection before trial medication administration to mitigate the risk of hypocalcaemia. If baseline serum vitamin D concentration is less than 30 nmol/L, an additional dose of cholecalciferol 50 000 IU is administered. Oral daily cholecalciferol 1000 IU is prescribed for 12 months. Oral cholecalciferol is withheld if the baseline serum vitamin D concentration is greater than 100 nmol/L (40 ng/mL). The trial schedule is provided in Fig. 2.

Participants complete a BMD assessment as soon as possible after initial trial medication administration, and again one year later, to assess the primary outcome. Participants aged 70 years or older with a T-score of −2.5 or less on baseline BMD or those with a newly diagnosed fragility fracture during the trial are informed of the result by the site investigator and offered the option to be unblinded and continue in the trial, remain blinded until completion, or withdraw from the trial.

Participants may receive standard concomitant care apart from antiresorptive therapies for the trial duration. At trial completion, a written summary of BMD results, antiresorptive therapy received, and guideline recommendations for ongoing treatment will be provided by central unblinded staff.

### 2.5 Outcomes

#### 2.5.1 Primary outcome

The primary outcome is annualised change in femoral neck BMD during the year after ICU discharge. This is calculated as the difference between BMD at baseline and at one year, expressed as a percentage of the baseline BMD. The annualised value is calculated by dividing the percentage change by the actual number of days between BMD assessments and then multiplying by 365. For clinical context, the absolute baseline and one-year BMD values will be included to provide assessment of baseline status and absolute change between groups.

#### 2.5.2 Secondary outcomes

Secondary outcomes include (i) annualised change in lumbar spine BMD during the year after critical illness; (ii) clinical fragility fracture (self-reported and confirmed by X-ray report) and vertebral fracture assessed using vertebral fracture assessment (VFA) on lateral Dual-energy X-ray Absorptiometry (DXA) scan; (iii) self-reported falls incidence and frequency at 6 and 12 months; (iv) hospital readmission during the trial; (v) mortality during the trial; and (vi) quality of life (EuroQol 5 Dimensions 5 Levels measured at baseline, 6 and 12 months). Hospital readmissions and mortality will be censored at day 90 for participant's lost to follow-up. Fracture risk assessment will be performed for each trial participant using the Australian version of the FRAX fracture risk assessment tool, an algorithm developed by the World Health organization (WHO) that combines clinical risk factors with or without femoral BMD to estimate 10-year probability of hip and major osteoporotic fracture.^16^

#### 2.5.3 Safety outcomes and adverse events

All adverse events considered potentially causally related to the trial intervention will be reported as adverse events or serious adverse events. Serious adverse events include: (i) severe hypocalcaemia (ionised calcium <0.70 mmol/L and/or serum calcium <1.55 mmol/L with symptoms of hypocalcaemia); (ii) osteonecrosis of the jaw (non-healing dental ulcer of >8 weeks' duration with exposed bone); (iii) new infection (skin erysipelas, cellulitis, abdominal, urinary tract, respiratory, bacteraemia, sepsis, or septic shock) defined as commencement of antibiotics for clinically suspected infection and at least one of temperature >38 °C, white cell count >11.0 × 10^9^/L, or positive culture; and (iv) new renal failure (using five-category scoring system to evaluate risk, injury, failure, loss, and end-stage kidney injury^17^). Adverse events include haematological, biochemical, and adverse clinical outcomes judged by the site investigator to be related to administration of trial medication.

### 2.6 Data collection, management, and closing of the database

Trained research coordinators collect data at each site, and these data are managed in a web-based case report form (Appendix 1). Data monitoring is performed according to a prespecified monitoring plan by a trained project manager and monitor from the Australian and New Zealand Intensive Care Research Centre. The database is locked when all data are entered and all discrepant or missing data are resolved, after all efforts are made to complete the database, and it is considered that remaining issues cannot be fixed. A review of data is planned before database lock. After review, the database is locked and exported for statistical analysis, permission for access to the database is removed, and the database is archived.

### 2.7 Data safety and monitoring board

The independent Data Safety and Monitoring Board (DSMB) comprises clinicians and a statistician experienced in the conduct, monitoring, and analysis of randomised clinical trials. An agreed DSMB charter is in place before commencement of recruitment. The role of the DSMB is to protect the safety of participants enrolled in the trial. The DSMB reviews all reported serious adverse events as they occur. Stopping rules pertaining to participant safety are not predefined, and the DSMB may recommend stopping the trial for patient safety at its discretion. The DSMB has discretion to request further information at the site level if required. The DSMB acts in an advisory capacity to the trial management committee, which is responsible for the conduct of the trial.

### 2.8 Initial sample size calculation and analysis plan

BONE ZONE was initially designed and commenced recruitment as a classical frequentist trial. To detect a clinically significant 2% absolute difference in annual change in femoral neck BMD with standard deviation of 4%, power of 90%, in a three-group sample to detect pairwise differences between any two of the three groups, a sample size of 108 per intervention group is required. Allowance for a predicted 15% mortality and 15% loss to follow-up resulted in an overall sample size of 450 participants. In May 2024, the DSMB performed a planned analysis after 130 participants were enrolled, and no safety issues were identified. The major issue raised was slow enrolment, contributed to by trial commencement during the COVID pandemic, with follow-up limited due to public health restrictions, prospective participant consent during ICU admission, and in-person follow-up. After meeting with the DSMB, the power was recalculated to 80% and the sample size was redefined as 330 participants. Further discussions with the DSMB in August 2024, without knowledge of any accumulated trial outcome data, led to the BONE ZONE management committee approving: (i) enrolment until the end of February 2026; and (ii) change to a Bayesian analysis as the primary trial analysis plan. There are no changes to other components of the trial design.

### 2.9 Primary analysis plan

Bayesian methods allow estimation of the probability of different magnitudes of treatment effect, which clinical researchers may interpret more easily than statements from conventional frequentist statistical models about rejection of a null hypothesis. Simulation studies of the primary outcome within this new Bayesian framework demonstrate adequate preservation of the original operating characteristics of the trial with respect to 90% power and 5% type I error (Appendix 2). This was demonstrated using 10 000 simulations of the trial conduct under the null and in different scenarios (Table 2), using the Core Adaptive Continuous Design Module within the Fixed and Adaptive Clinical Trial Simulator (FACTS) software (FACTS Development Team 2023, Austin, TX, USA).

**Table 2:** Operational characteristics of different scenarios and assumptions (probability ≥97.5%).

	Type I Error	Power	Futility	Inconclusive
Null scenario	0	–	1	0
Scenario 1: ≥2% increase in any arm vs. 0% change in control	–	0.04	0.32	0.628
Scenario 2: ≥2% increase in zoledronic acid vs. 0% change in control and denosumab	–	1.00	0.00	0.00
Scenario 3: ≥2% increase in denosumab vs. 0% change in control and zoledronic acid	–	1.00	0.00	0.00
Scenario 4: ≥2% increase in any arm vs. 2% decline in control	–	0.99	0.00	0.01
Scenario 5: ≥2% increase in zoledronic acid vs. 2% decline in control and 0% change in denosumab	–	0.96	0.00	0.04
Scenario 6: ≥2% increase in denosumab vs. 2% decline in control and 0% change in zoledronic acid	–	0.96	0.00	0.04
Scenario 7: ≥4% increase in denosumab vs. 0% change in control and zoledronic acid	–	0.96	0.00	0.04
Scenario 8: ≥4% increase in denosumab vs. 2% decline in control and 0% change in zoledronic acid	–	1.00	0.00	0.00
Scenario 9: ≥4% increase in zoledronic acid vs. 0% change in control and denosumab	–	0.96	0.00	0.04
Scenario 10: ≥4% increase in zoledronic acid vs. 2% decline in control and 0% change in denosumab	–	1.00	0.00	0.00
Scenario 11: ≥4% increase in any arm vs. 0% change in control	–	0.99	0.00	0.01
Scenario 12: ≥4% increase in any arm vs. 2% decline in control	–	1.00	0.00	0.00

Assumptions:1. Sample size: 330 patients2. Time to final endpoint: 48 weeks3. Standard deviation of the response: 0.044. No dropouts5. Clinically significant difference: 0.026. Posterior probability: Pr(θ_d - θ_(Control) > 0.02)7. Decision: Pr(θ_d - θ_(Control) > 0.02); d = greatest Pr(EDq relative to control: quantile 0.975)8. Futility criterion: Pr(θ_d - θ_(Control) > 0.02); d = greatest Pr(EDq relative to control: quantile 0.975) < 0.509. Superiority criterion: Pr(θ_d - θ_(Control) > 0.02); d = greatest Pr(EDq relative to control: quantile 0.975) > 0.97510. Weakly informative priors for all arms (mean [μ_0d_] 0 and sd [v_0d_] 0.20). Mean was set to 0 because this is the expected response on the control arm and the standard deviation to 0.20 because this is five times the expected standard deviation of the response11. Weakly informative sigma priors (alpha 0.5 [central value 0.04] and beta 0.0008 [weight 1]). Alpha was set to 0.5 because the central value was set to the same expected standard deviation of the response and the number of observations weight for the prior to 1 (so the prior is weak, equivalent to a single subject's responses and the impact of the prior on the final posterior will be negligible).

### 2.10 Statistical analyses

All statistical analyses will be conducted on an intention-to-treat basis, with analysis according to assigned treatment arm. Complete case analysis will be carried out for all the outcomes. If missing data are found for the primary outcome, analyses using multiple imputation will be carried out for survivors. Multiple imputation will consider models based on prognostic baseline and post-baseline variables under a missing-at-random assumption. All analyses will be performed using R v.4.3.3 (Vienna, Austria). Participants who die before the one-year BMD assessment will not contribute to the primary outcome analysis because the outcome cannot be measured post mortem. Deaths will be recorded and reported as part of the secondary outcomes, but no imputation for deaths will be made for the primary endpoint. The expected mortality rate was accounted for in the original sample size calculation to preserve statistical power for survivors with evaluable BMD data.

The primary outcome in each of the treatment groups will be modelled independently with the use of Bayesian analysis, and all primary models described below will be adjusted only for the stratification variables (sex and site) as random effects. All Bayesian models will be fitted with the integrated nested Laplace approximation (INLA), allowing the calculation of posterior effect estimates with their 95% credible intervals (CrI) and the probability that each treatment is the most or least effective.

The threshold for superiority and futility will be defined at the treatment-comparison level. Three posterior probabilities will be drawn to calculate the probability that a given treatment is better or worse than the other two. The criterion for declaring the most or least effective treatment will be a probability greater than 0.975. Specifically, if the posterior probability that one treatment is the most effective exceeds 0.975, then that treatment will be declared superior to the other two. This threshold mirrors a one-sided frequentist alpha of 0.025 and ensures that only one treatment can be identified as “best.” Using the same threshold, if the posterior probability that a treatment is the least effective exceeds 0.975, then that treatment will be declared inferior. This is the Bayesian analogue to futility stopping in conventional trials. If none of the treatments cross the 0.975 threshold for best or worst, no treatment will be formally declared superior or inferior. Instead, posterior probabilities and 95% credible intervals will be reported, allowing readers to interpret the relative likelihood of benefit or harm.

The primary outcome will be analysed with a Bayesian linear regression model, which will calculate posterior probability distributions of mean annualised change in femoral neck BMD based on evidence accumulated in the trial and the prior probability distribution (the assumed previous knowledge). Other continuous outcomes will be analysed using the same model described for the primary outcome. Binary outcomes will be reported using a Bayesian logistic regression model, expressed as relative risks with their 95% confidence intervals. Time-to-event outcomes will be analysed using a Bayesian Cox proportional hazards model, reported as hazard ratios with their 95% CrI.

#### 2.10.1 Priors

**Fig. 3:**
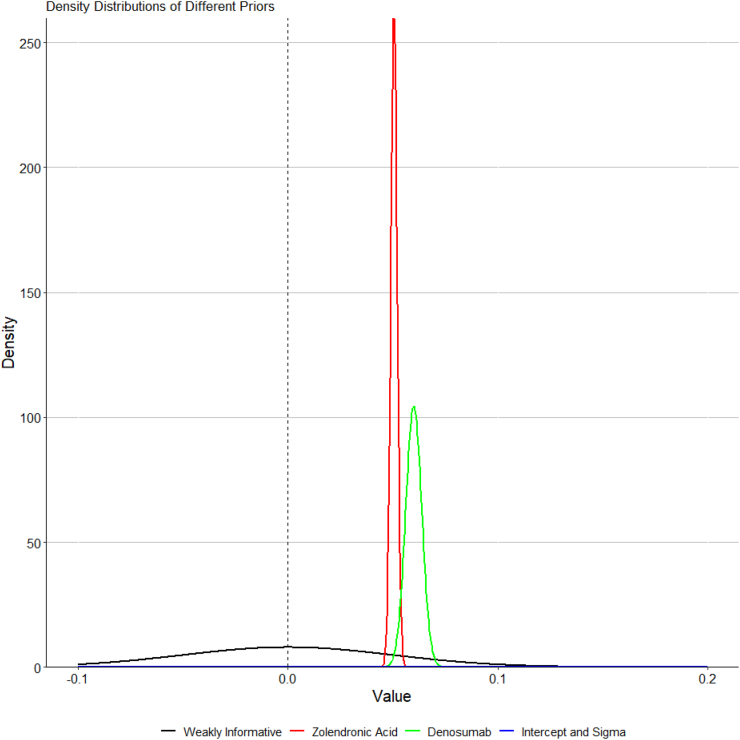
Prior distributions.

Prior distributions for individual treatment effects for all analyses described above will be neutral (weakly informative) (Fig. 3). A prior of Normal(0, 0.05) will be used for all treatment arms. The prior is centred at 0, reflecting no expected effect a priori, and the standard deviation of 0.05 provides weak regularisation, allowing the data to dominate the posterior distribution. The Intercept and residual standard deviation (sigma) priors will be weakly informative and defined as *t*(3, 0, 2.5).

#### 2.10.2 Sensitivity analyses

A sensitivity analysis using informative priors will be performed. These priors will be based on previous evidence on the impact of zoledronic acid and denosumab on annualised change in femoral neck or total hip BMD. The informative prior for the zoledronic acid will be estimated from a previous study showing an increase in BMD in femoral neck after one year in postmenopausal women of 5.1% (95% CI, 4.8–5.4) compared to placebo.^5^ This will translate to a prior Normal(0.0506, 0.00153) (Fig. 3). The informative prior for the denosumab will be estimated from a previous study showing an increase in BMD in total hip after 36 months in postmenopausal women of 6.0% (95% CI, 5.2–6.7) compared with placebo.^4^ This will translate to a prior Normal(0.0600, 0.00382) (Fig. 3).

Two additional sensitivity analyses will be performed: (i) assessment of the primary outcome considering additional adjustment for potential imbalances in baseline characteristics; and (ii) additional models comparing “intervention” vs. placebo, where intervention is denosumab and zoledronic acid combined.

#### 2.10.3 Subgroup analysis

Subgroup analyses will be conducted to explore the consistency of the treatment effect across the following pre-defined subgroups: (i) sex and (ii) baseline frailty status (Clinical Frailty Scale 1–3 [non-frail] vs. 4–5 [mild frailty] vs. 6–8 [moderate to severe frailty]). It is hypothesised that efficacy will differ within these subgroups, with an increased treatment effect observed in women and with increased baseline frailty. For each subgroup, the primary outcome will be analysed using Bayesian linear regression models including an interaction term between the treatment group (zoledronic acid, denosumab, placebo) and the subgroup variable of interest. The posterior probability that the treatment effect differs across subgroup categories will be assessed to evaluate potential effect modification. These analyses are exploratory and will be interpreted with caution given the sample size and multiplicity of comparisons.

## 3 Trial reporting and data sharing

Trial results are to be shared through peer-reviewed publications and plain language summaries. De-identified individual participant data will be made available in accordance with the Australian and New Zealand Intensive Care Research Centre Data Sharing Policy.

## 4 Summary

BONE ZONE is a phase IIb, multicentre, prospective, double-blind, placebo-controlled, randomised trial comparing the safety and efficacy of zoledronic acid or denosumab with placebo in a population of patients recovering from critical illness. The primary outcome is the annualised change in femoral neck BMD for the year after ICU discharge. The protocol and statistical analysis plan were submitted for publication before recruitment was complete. As of 15 December 2025, 231 participants had been enrolled in the trial.

## CRediT authorship contribution statement

NO - conceptualisation, writing (original draft), writing (review and editing).

AS - conceptualisation, writing (original draft), writing (review and editing), methodology, formal analysis

PN - conceptualisation, writing (review and editing).

AB - conceptualisation, writing (review and editing).

JC - writing (review and editing).

CH - writing (review and editing).

MK - writing (review and editing).

EL - writing (review and editing).

CR - writing (review and editing).

TT - writing (review and editing).

PY - writing (review and editing).

BV - writing (review and editing).

## Funding

This work was supported by the Medical Research Future Fund Rare Cancer Rare Disease Unmet Need Project Grant (RCRDUN 2019 APP1199726). The funding body had no input into the design or conduct of the trial, statistical analysis plan and analysis, or reporting of results.

## Conflict of interest

The authors declare the following financial interests/personal relationships which may be considered potential competing interests: Neil Orford reports financial support was provided by the Medical Research Future Fund. If there are other authors, they declare they have no known competing financial interests or personal relationships that could have influenced the work reported in this paper. Ary Serpa Neto and Paul Young are Acting Editors-in-Chief, Carol Hodgson is an Associate Editor and Ed Litton and Balasubramanian Venkatesh are on the Editorial Board with Critical Care and Resuscitation. Acting Editors-in-Chief Ary Serpa Neto and Paul Young were not involved in the peer review of the paper or overseeing the peer review process.
