# Supplementary material for: Protocol summary and statistical analysis plan for the bone loss prevention with zoledronic acid or denosumab in critically ill adults (BONE ZONE) trial
Source: Crit Care Resusc. 2026 Mar 24;28(2):100173. doi: 10.1016/j.ccrj.2026.100173 (PMC13050062; doi:10.1016/j.ccrj.2026.100173)
Supplement: Multimedia component 1 [file mmc1.docx]

**Protocol summary and statistical analysis plan for the bone loss prevention with zoledronic acid or denosumab in critically ill adults (BONE ZONE) trial**

ONLINE SUPPLEMENT

CONTENT

Appendix 1 – BONE ZONE Case Report Form

Appendix 2 – BONE ZONE Simulation Studies

Appendix 3 – BONE ZONE Investigators

**Protocol summary and statistical analysis plan for the bone loss prevention with zoledronic acid or denosumab in critically ill adults (BONE ZONE) trial**

APPENDIX 1


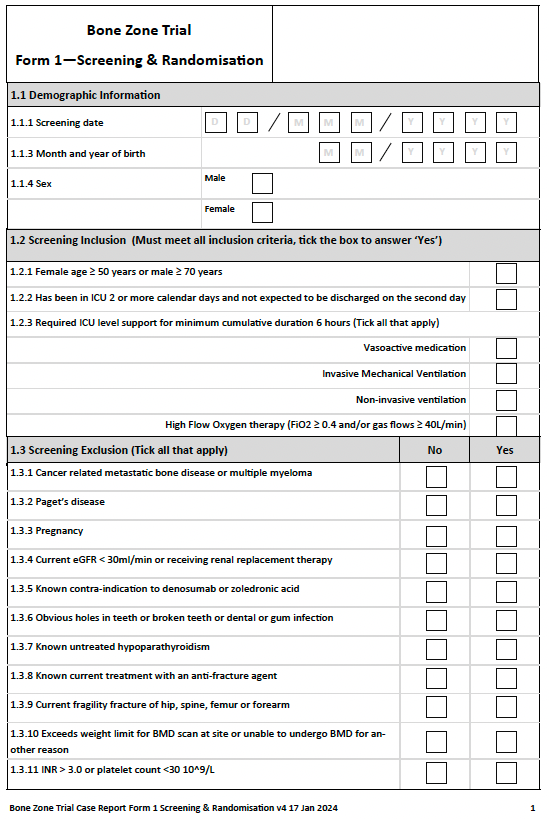


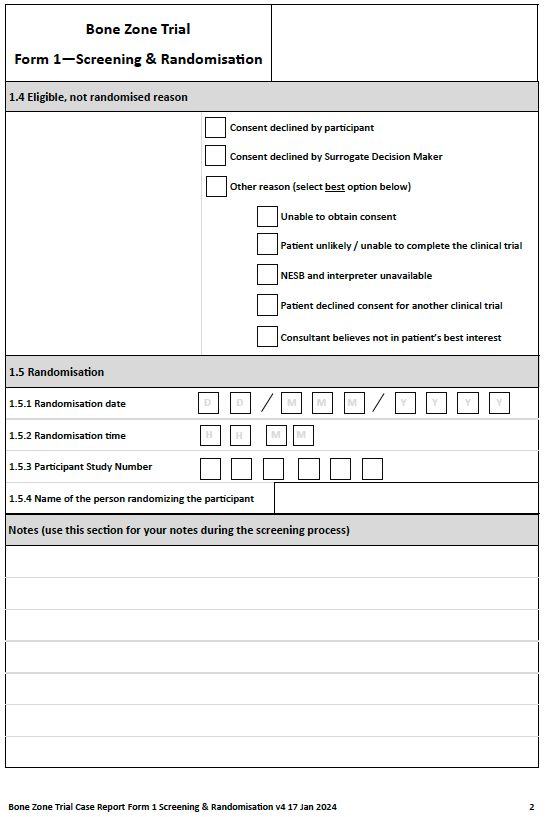


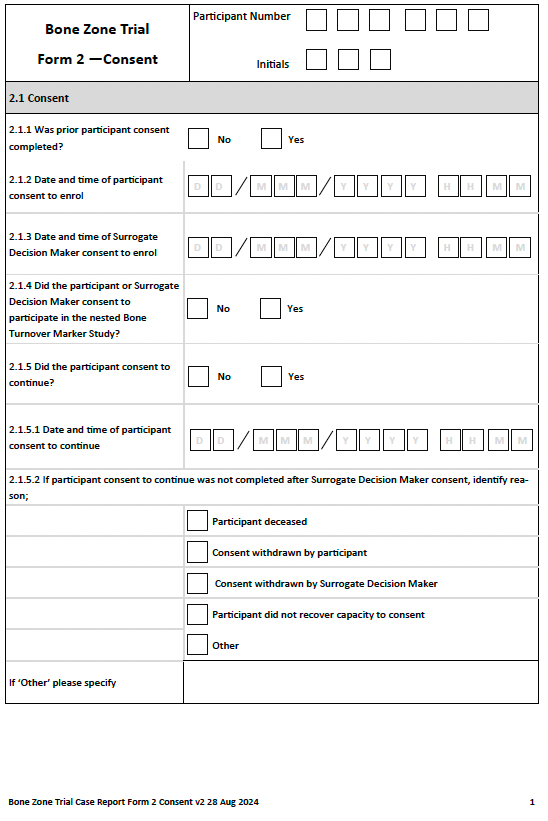


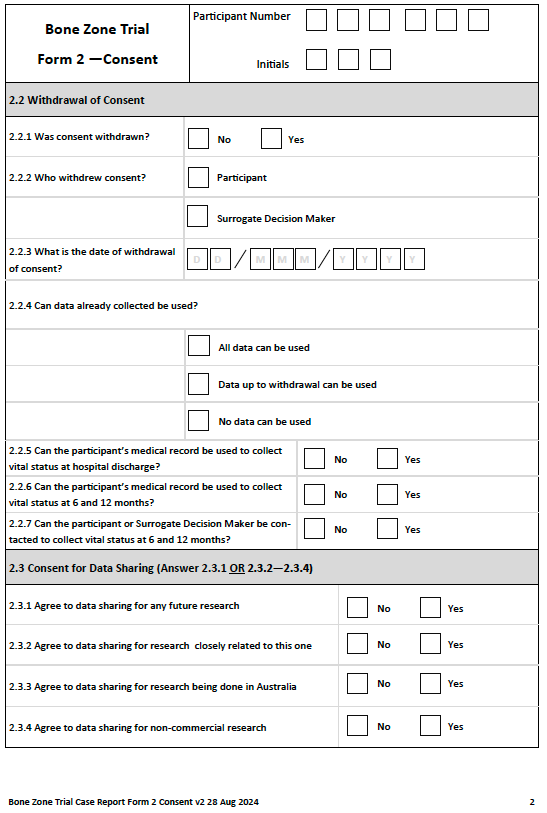


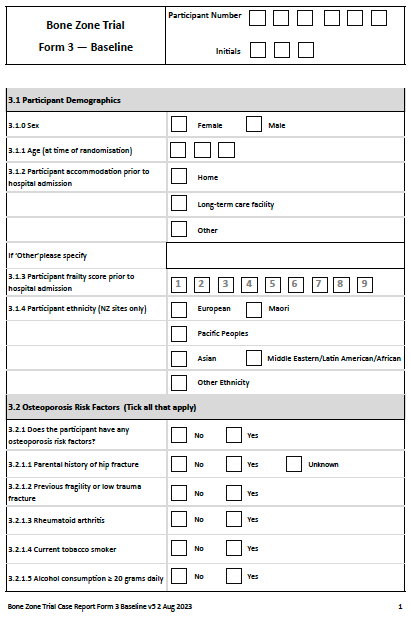


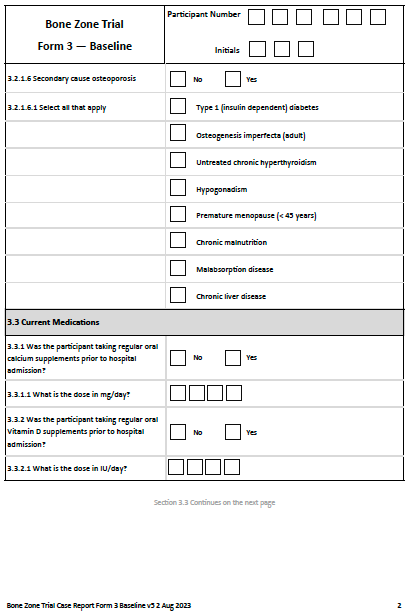


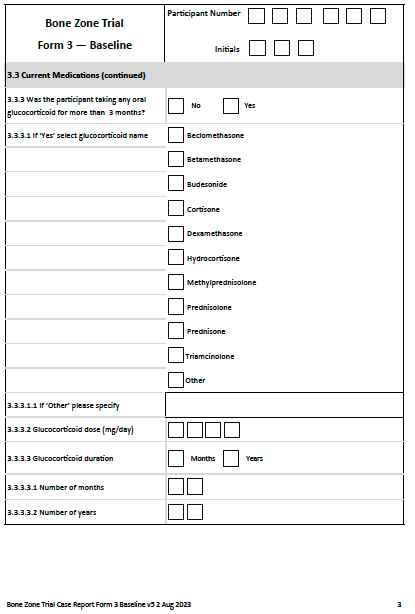


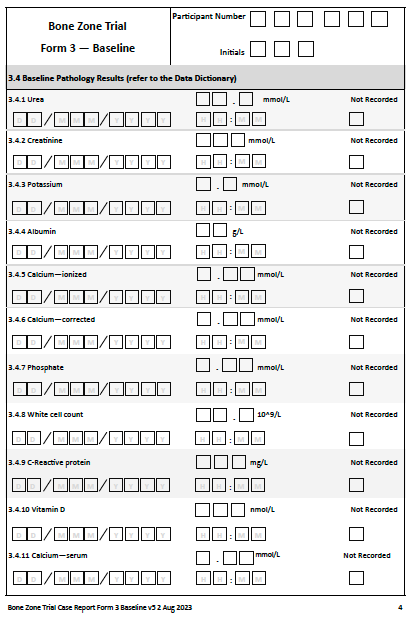


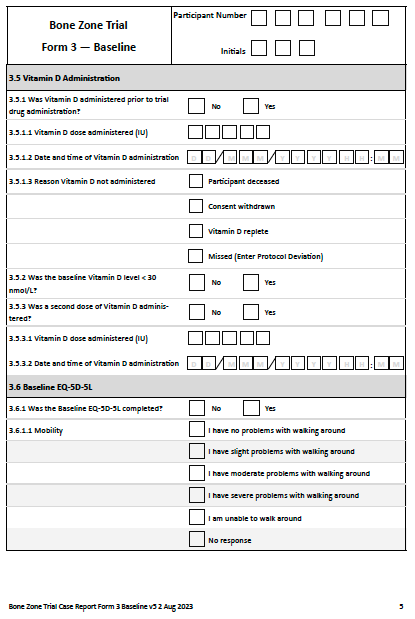


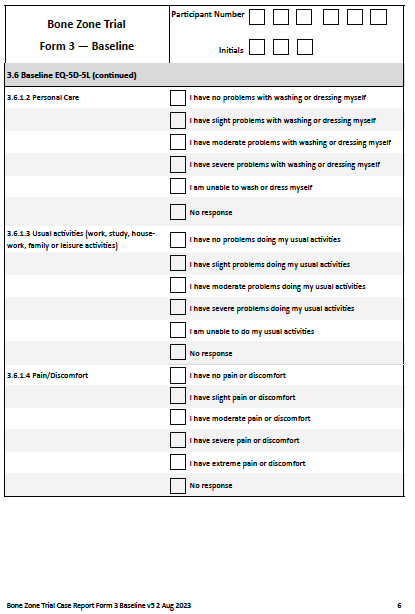


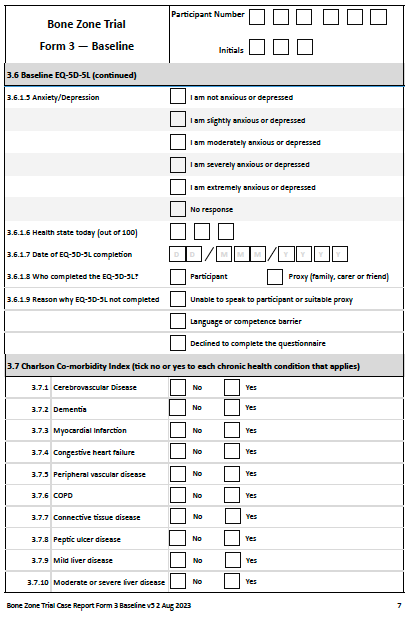


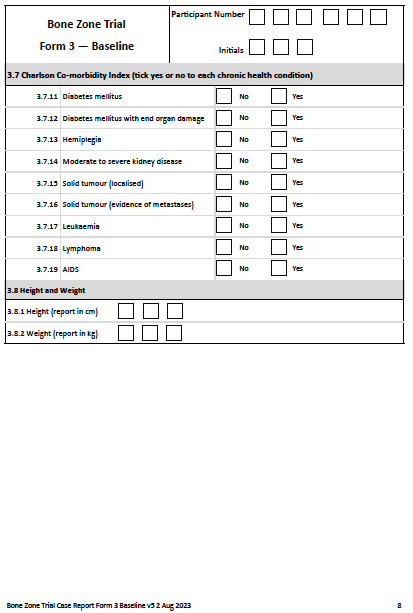


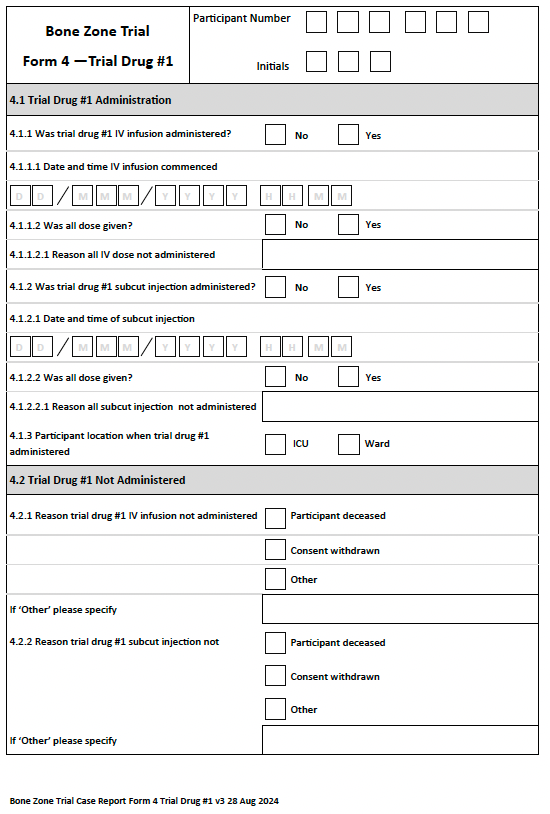


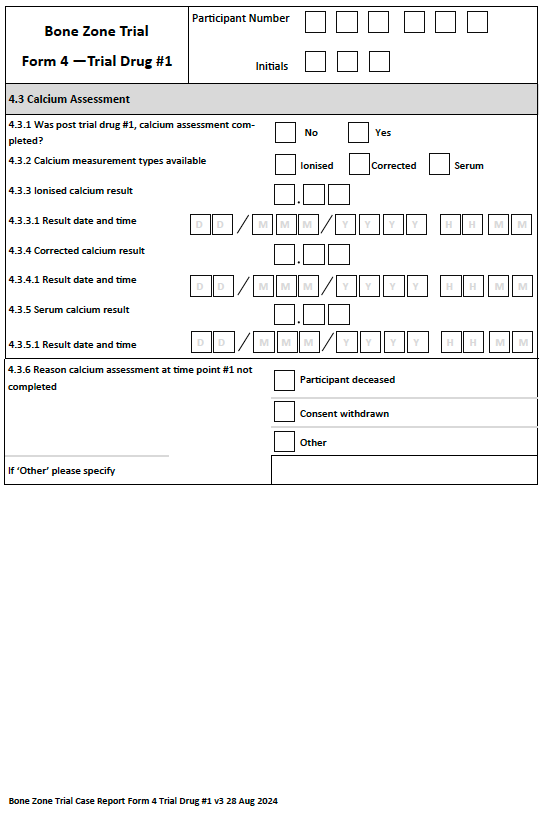


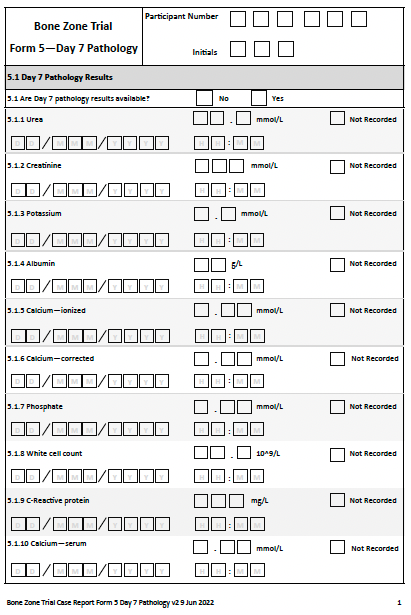


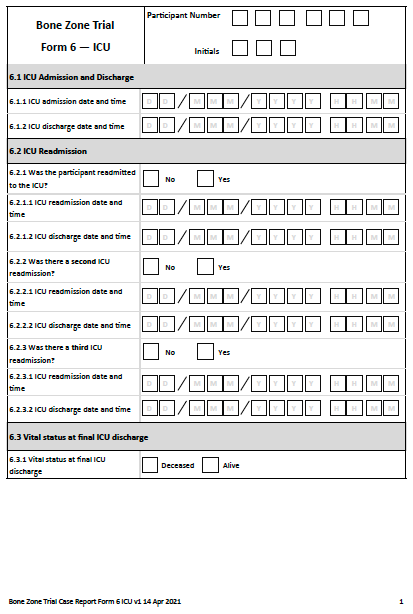


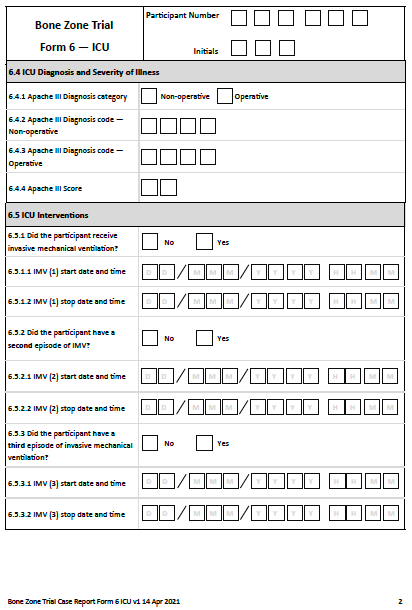


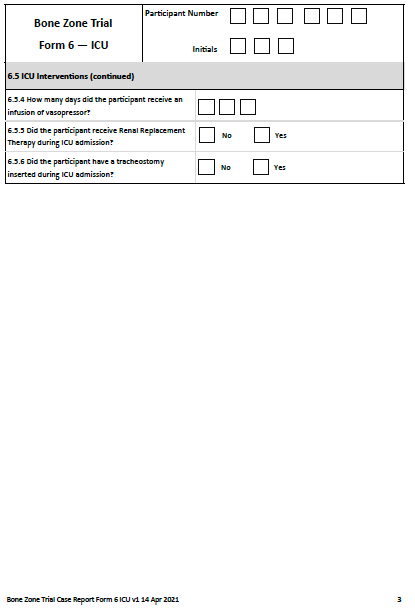


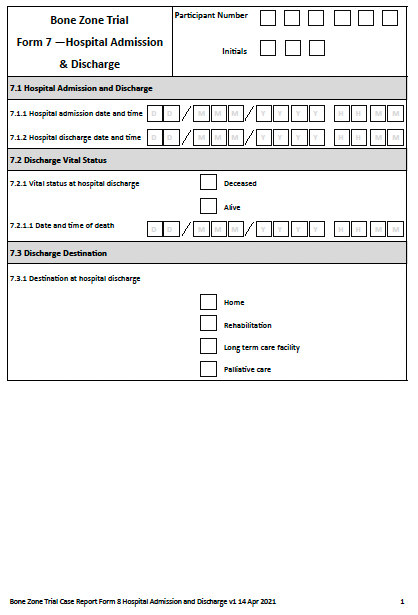


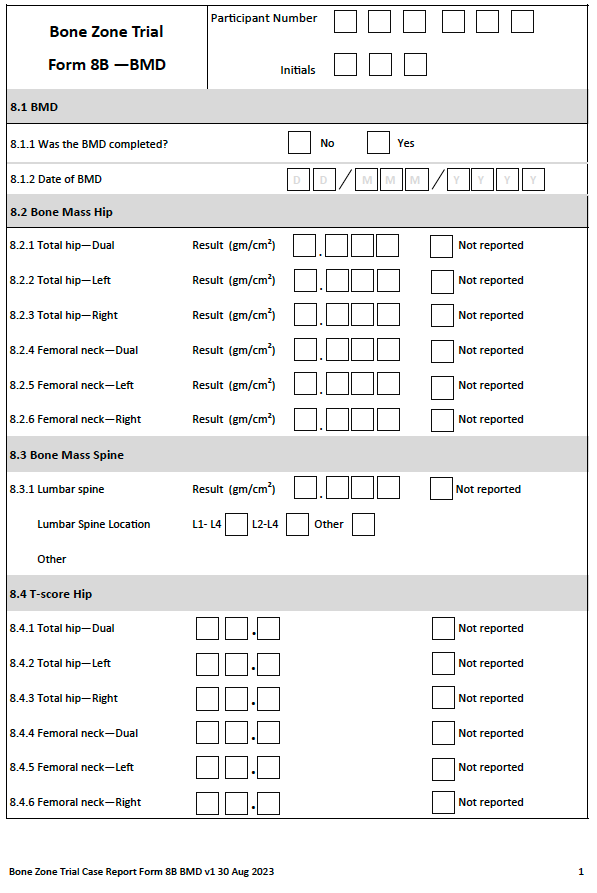


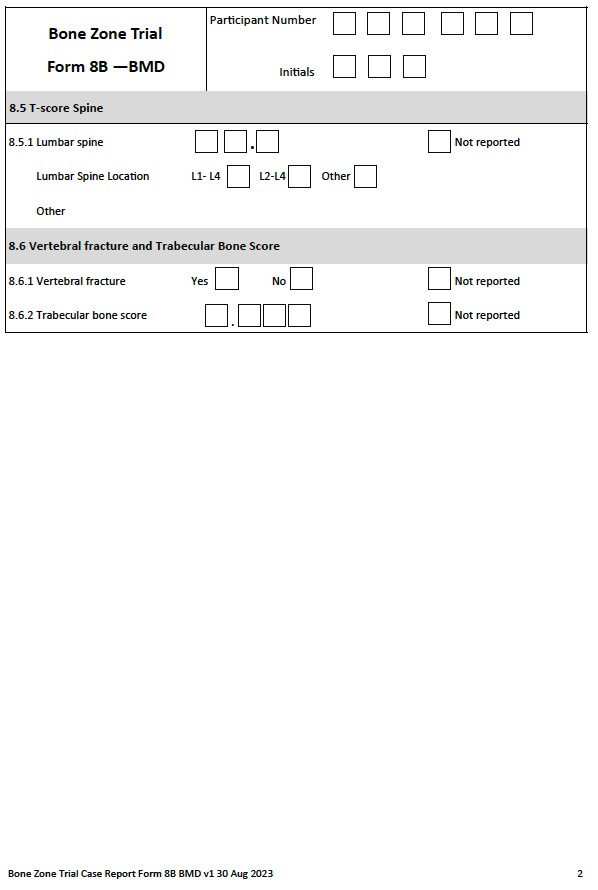


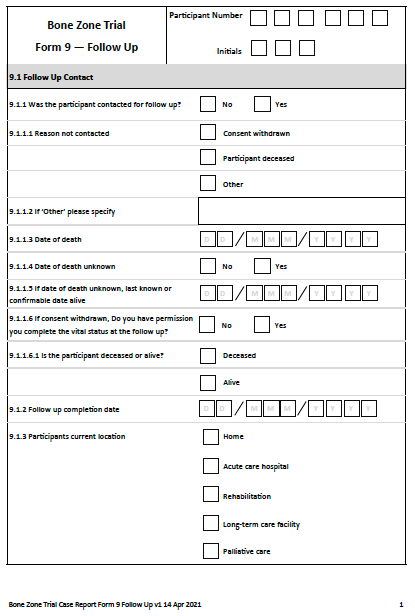


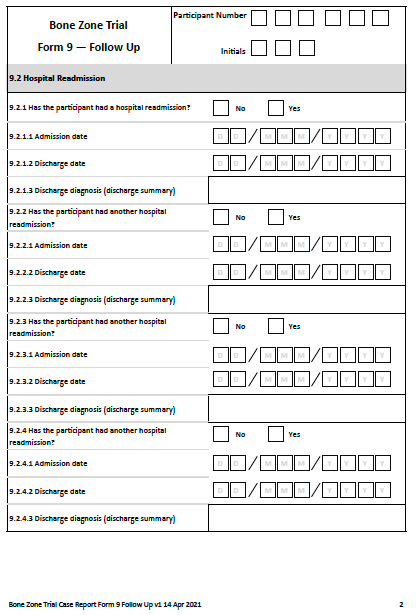


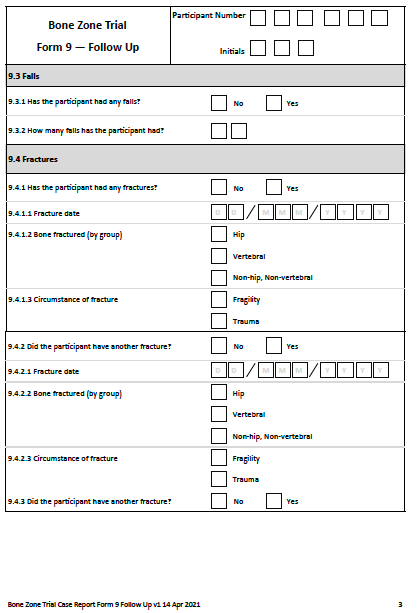


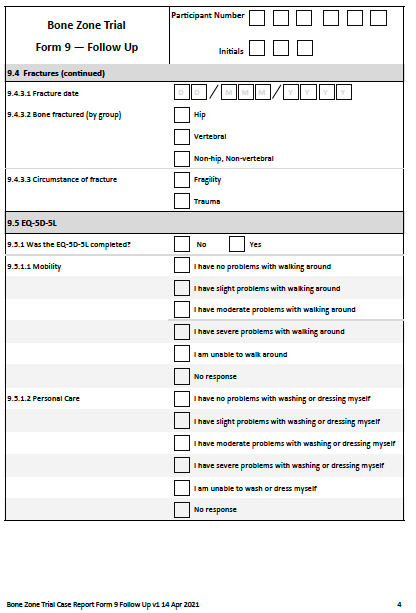


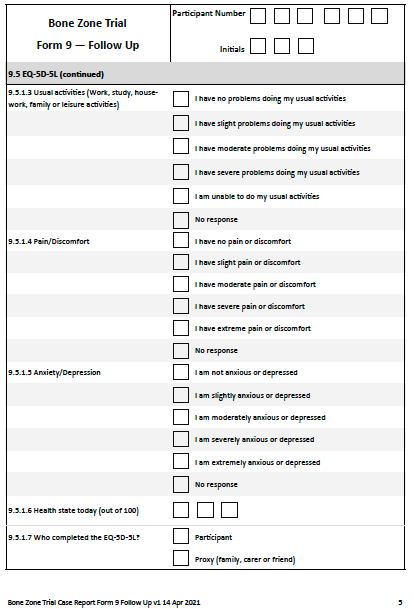


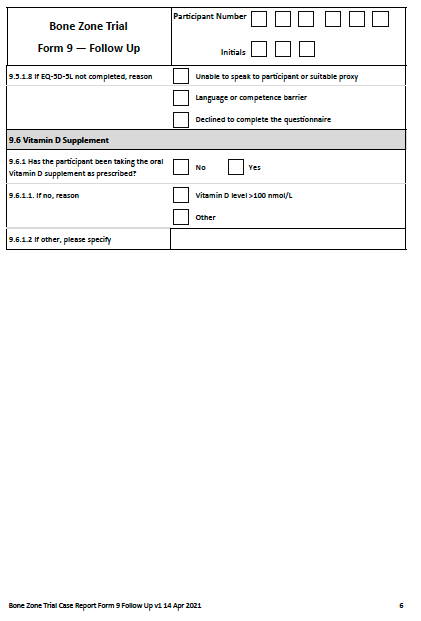


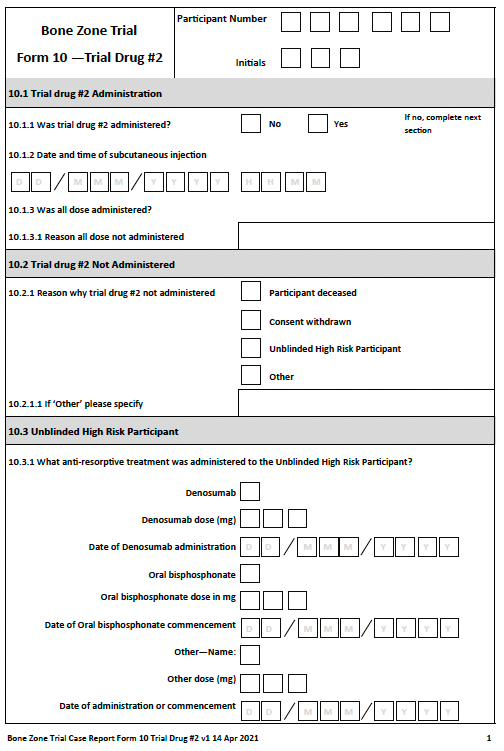


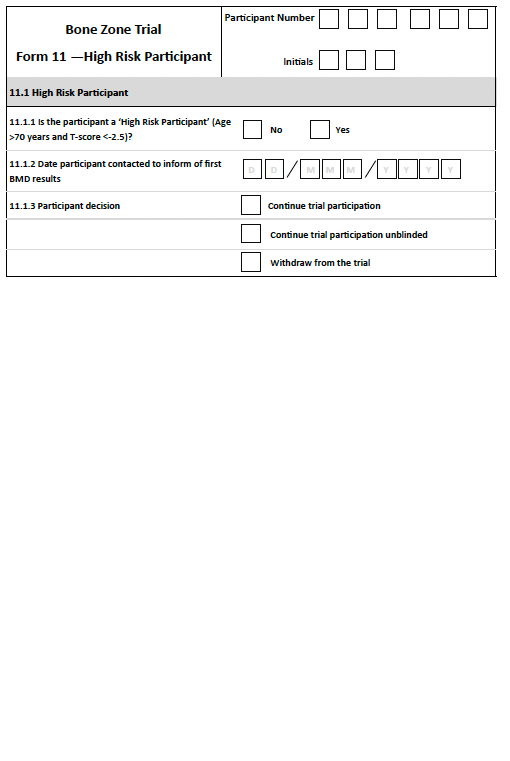


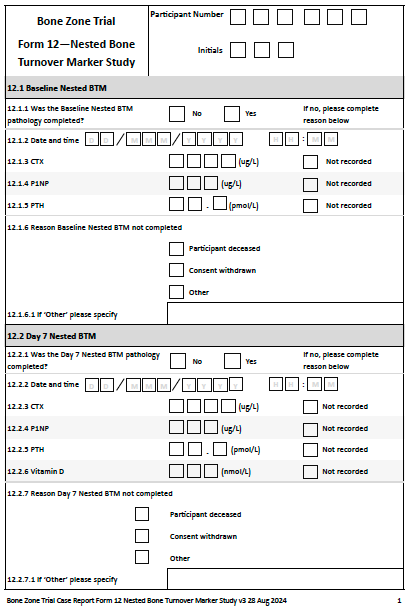


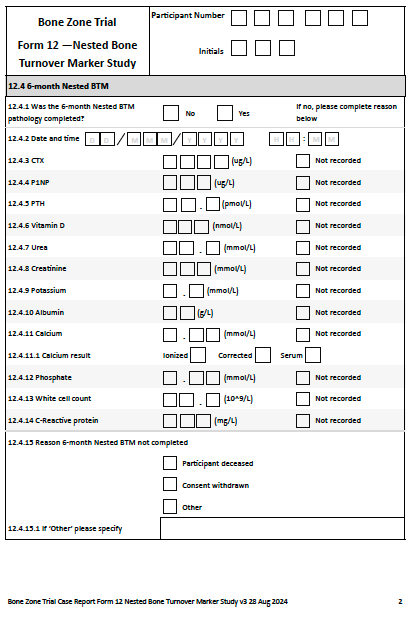


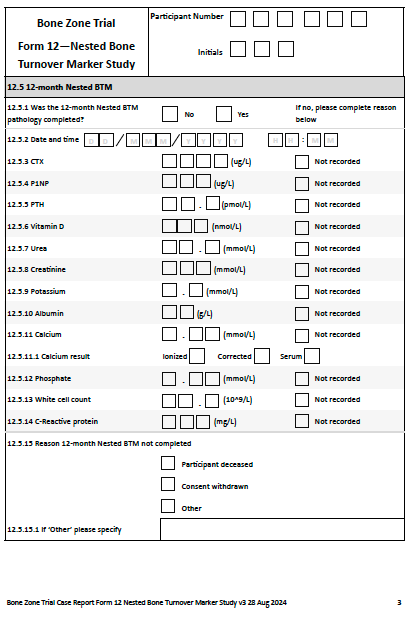


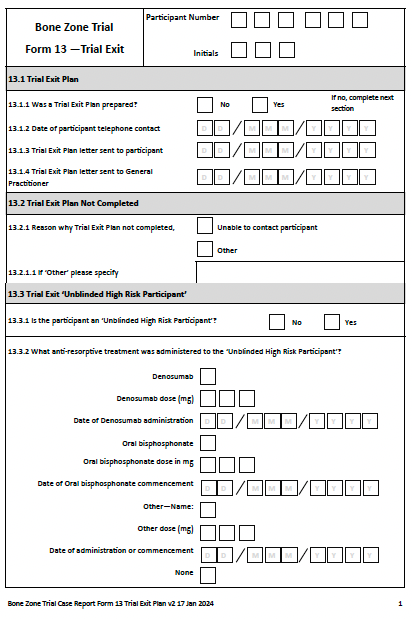


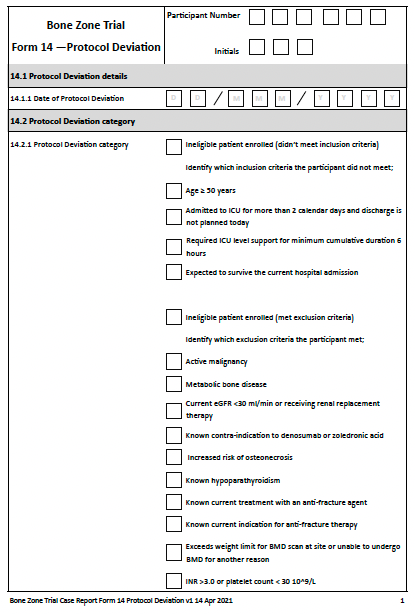


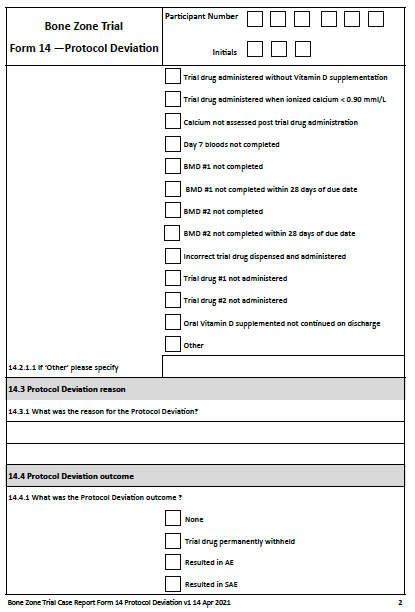


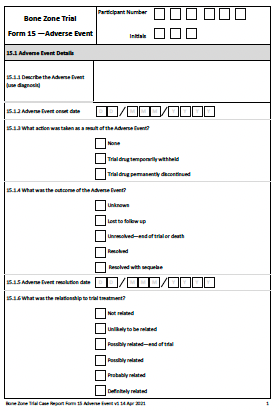


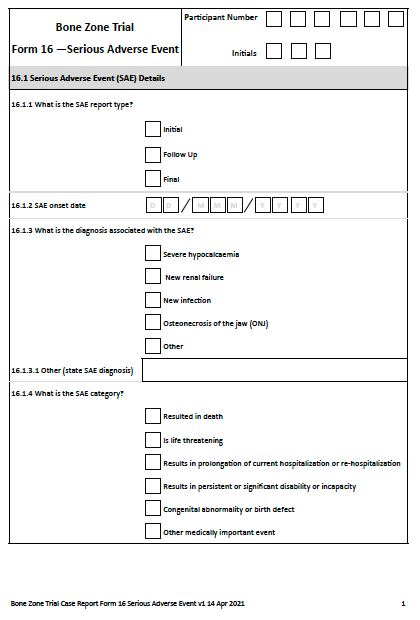


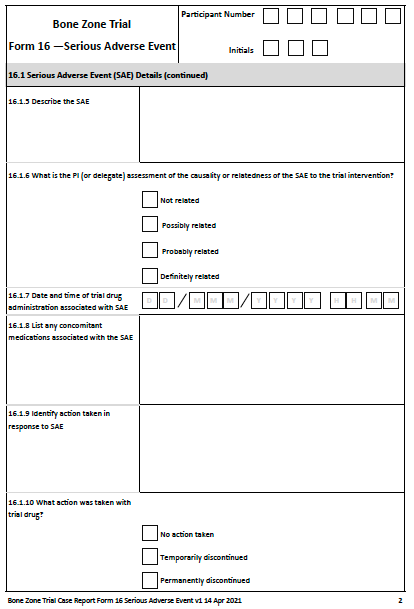


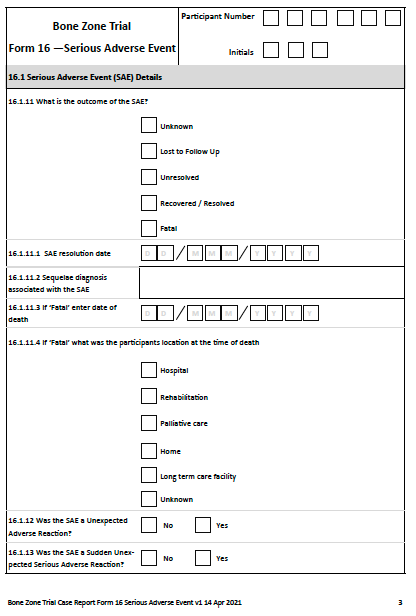


**Protocol summary and statistical analysis plan for the bone loss prevention with zoledronic acid or denosumab in critically ill adults (BONE ZONE) trial**

APPENDIX 2


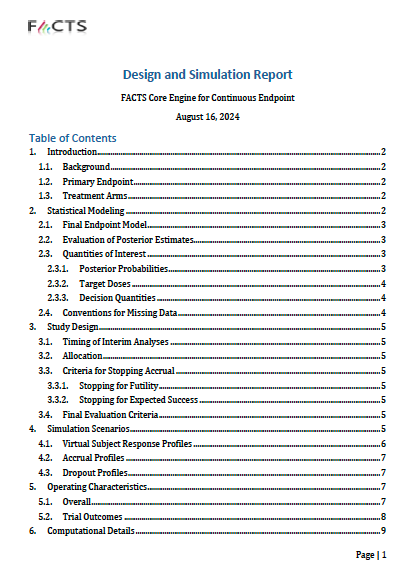


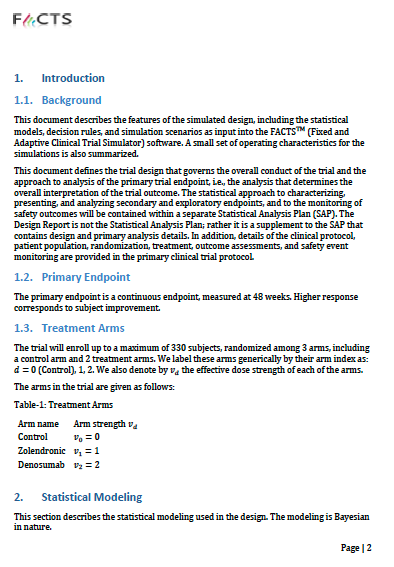


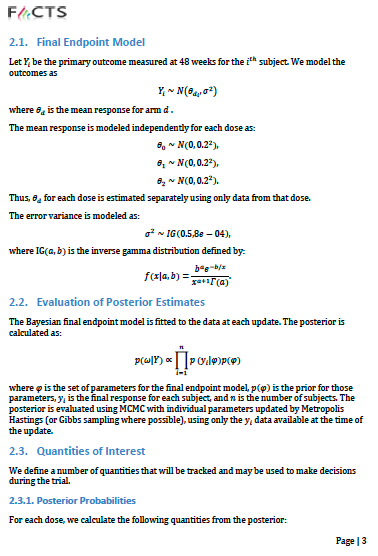


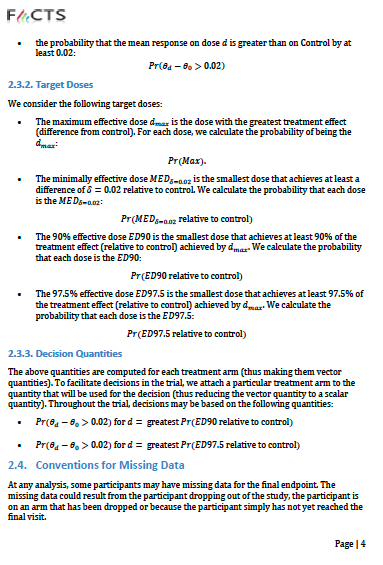


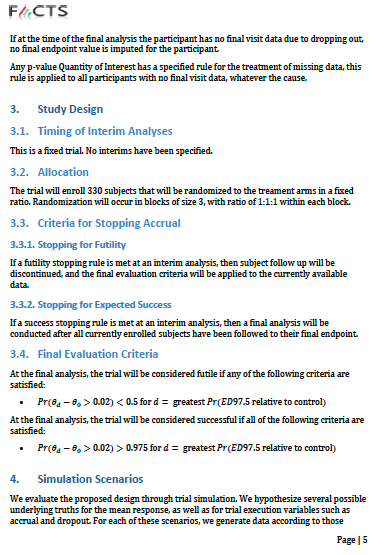


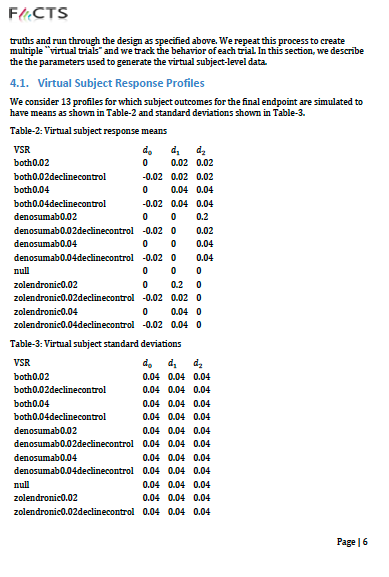


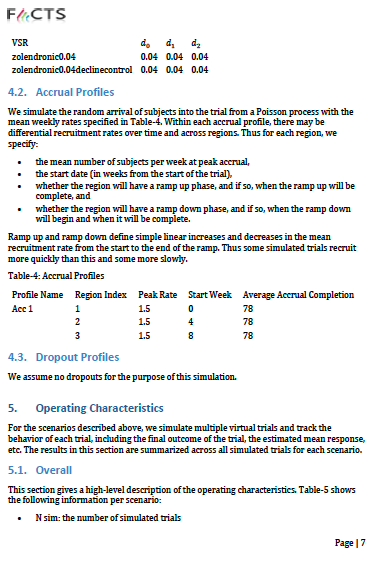


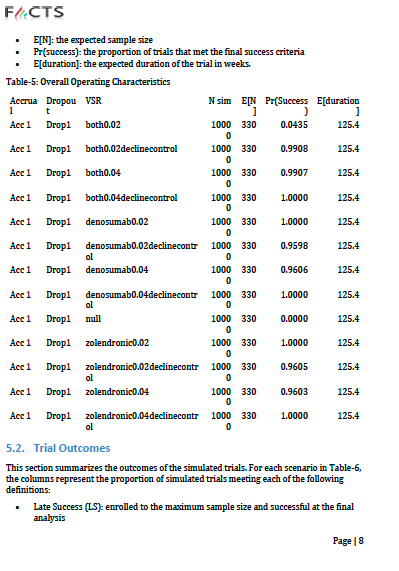


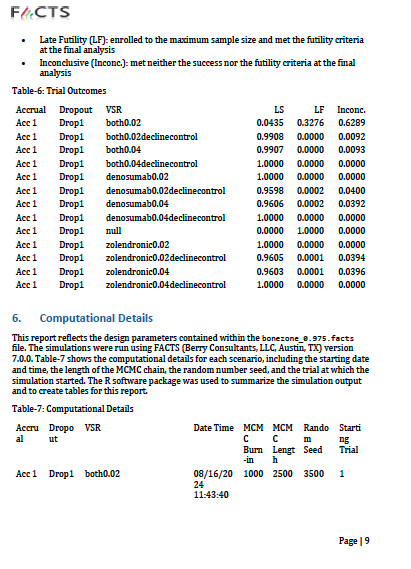


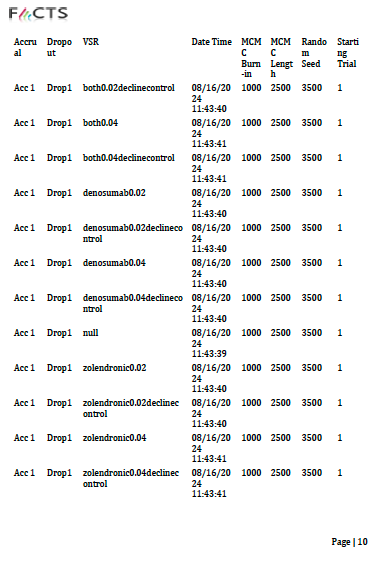


**Protocol summary and statistical analysis plan for the bone loss prevention with zoledronic acid or denosumab in critically ill adults (BONE ZONE) trial**

APPENDIX 3

| **Institution** | **Principal Investigators** | **Research Coordinators** |
| --- | --- | --- |
| University Hospital Geelong | A/Prof Neil Orford | Simone Fitzgerald, Jemma Trickey, Stacey Hawker |
| Austin Hospital | Prof Ary Serpa Neto | Glenn Eastwood, Helen Young, Leah Peck |
| Alfred Hospital | Dr Elisa Licari | Emma Martin, Jasmin Board |
| St Vincent’s Hospital Melbourne | Prof John Santamaria | Jennifer Holmes, Viean Luk, Melissa King |
| Eastern Health | Dr Paul Rob | Nicole Robertson, Kym Gellie |
| Western Health | Prof Craig French | Samantha Bates, Miriam Towns, Stephanie Menadue |
| Royal Melbourne Hospital | Dr Emily See | Kathleen Byrne, Deborah Barge |
| Peninsula Health | Dr Ravi Travindranath | David Zhang |
| St Vincent’s Hospital Sydney | A/Prof Priya Nair | Amelia Hall, Alexandra Campion, Amelia Liu, Sarah Henstridge |
| Royal Prince Alfred | A/Prof David Gattas | Heidi Buhr, Cindy Liang, Bronwyn Kelly |
| John Hunter Hospital | Dr Rakshit Panwar | Amber Poulter, Sarah Dalton |
| Illawarra Shoalhaven Health | Dr Ahmad Elgendy | Wenli Geng |
| Prince of Wales | Dr Gordon Flynn | Raymond Lewis |
| St George Hospital | Dr Manoj Saxena | Chloe Edwards, Sarah Valle Fitzsimmons, Rebecca Sidoli |
| Blacktown Hospital | Dr Yi-Lun Tsai | Treena Sarah |
| Wesley Hospital | Prof Jeremy Cohen | Shaoning LV, Amanda Davie, Alison Leech, Amy Owens |
| Gold Coast University Hospital | Dr James Winearls | Mandy Tallott, Maree Houbert, Julie Pitman |
| Sunshine Coast Hospital | Dr Peter Garrett | Jane Brailsford, Lauren Murray |
| Royal Adelaide Hospital | Dr Mark Plummer | Kathleen Glasby, Sarah Doherty, Nerissa Brown, Paola Arce-Arango, Connor Christie |
| Launceston Hospital | Dr Matthew Brain | Jo Chen |
| SJOG Murdoch | A/Prof Adrian Regli | Annamaria Palermo |
| SJOG Subiaco | Dr Ed Litton | Janet Ferrier |
| Fiona Stanley Hospital | Dr Ed Litton | Annamaria Palermo |
| Wellington Hospital | Dr Paul Young | Lean Navarra |
| Auckland City Hospital | Dr Shae McGuiness | Rachael Parke, Magdalena Butler, Keri Anne Cowdrey, Alix Gray |
